# Mental Stress Classification Based on Selected Electroencephalography Channels Using Correlation Coefficient of Hjorth Parameters

**DOI:** 10.3390/brainsci13091340

**Published:** 2023-09-18

**Authors:** Ala Hag, Fares Al-Shargie, Dini Handayani, Houshyar Asadi

**Affiliations:** 1 School of Computer Science & Engineering, Taylor’s University, Jalan Taylors, Subang Jaya 47500, Selangor, Malaysia; s222327138@deakin.edu.au; 2 Institute for Intelligent Systems Research and Innovation, Deakin University, Geelong, VIC 3216, Australia; 3 Department of Electrical Engineering, Abu Dhabi University, Abu Dhabi P.O. Box 59911, United Arab Emirates; dinihandayani@iium.edu.my; 4 Computer Science Department, KICT, International Islamic University Malaysia, Kuala Lumpur 53100, Selangor, Malaysia

**Keywords:** channel selection, EEG, Hjorth parameters, machine learning, stress recognition

## Abstract

Electroencephalography (EEG) signals offer invaluable insights into diverse activities of the human brain, including the intricate physiological and psychological responses associated with mental stress. A major challenge, however, is accurately identifying mental stress while mitigating the limitations associated with a large number of EEG channels. Such limitations encompass computational complexity, potential overfitting, and the prolonged setup time for electrode placement, all of which can hinder practical applications. To address these challenges, this study presents the novel CCHP method, aimed at identifying and ranking commonly optimal EEG channels based on their sensitivity to the mental stress state. This method’s uniqueness lies in its ability not only to find common channels, but also to prioritize them according to their responsiveness to stress, ensuring consistency across subjects and making it potentially transformative for real-world applications. From our rigorous examinations, eight channels emerged as universally optimal in detecting stress variances across participants. Leveraging features from the time, frequency, and time–frequency domains of these channels, and employing machine learning algorithms, notably RLDA, SVM, and KNN, our approach achieved a remarkable accuracy of 81.56% with the SVM algorithm outperforming existing methodologies. The implications of this research are profound, offering a stepping stone toward the development of real-time stress detection devices, and consequently, enabling clinicians to make more informed therapeutic decisions based on comprehensive brain activity monitoring.

## 1. Introduction

According to recent neurosciences, the human brain is often considered the main target organ of mental stress due to its responsibility for distinguishing between situational circumstances (stressful/threatening or normal situations) [1]. To study the changes in brain activities during stress conditions, several non-invasive neuroimaging modalities have been used, such as functional magnetic resonance imaging (fMRI), positron emission tomography (PET), magnetoencephalography (MEG), electroencephalography (EEG), and functional near-infrared spectroscopy (fNIRS) [2,3]. EEG is a widely preferred modality for assessing brain functionalities due to its non-invasive nature, high temporal resolution, ease of setup, commercial availability, and comparatively low cost [4]. Accordingly, researchers use EEG in various domains that involve neural engineering, neurosciences, and biomedical sciences (e.g., brain–computer interfaces, BCIs) [5,6]. EEG signal plays a crucial role in several EEG-based research and application areas such as clinical applications for epilepsy [7], depression [8,9], the effective monitoring of emotion [10], mental stress [11,12,13], and sinogram [14].

Multiple EEG channels are often used for brain signal acquisition from multiple locations on the scalp to offer both high temporal and spatial resolutions. However, reducing the number of channels in the signal processing setup is necessary since the setup procedure with a high number of channels is time-consuming and can result in subject discomfort. Furthermore, it increases the system’s computational complexity, which is required to be low in specific applications [15,16]. Therefore, channel selection methods play a vital role in the reduction in complexity and high dimensionality of the feature vector space to improve overall performance. This increases the chances of building commercial wearable devices to provide a better diagnosis and accurate treatment for mental stress [17,18].

The common approach to channel selection methods is based on neuroscience skills where data from each region of the brain are highly correlated to some specific tasks. For example, the prefrontal region of the brain is highly associated with cognitive processing such as emotions, thoughts, and actions [2]. Meanwhile, central lobes relate to motor imagery tasks in BCI systems [15]. Consequently, several EEG channel selection methods based on specific tasks have been proposed [19,20] such as sequential floating forward selection (SFFS) in BCIs [21], normalized mutual information selection (NMIS) and minimum redundancy maximum relevance (mRMR) [22] in emotion recognition, spatiotemporal-filtering-based feature selection [20] in BCIs, and harmony search algorithm for alcoholism detection [23]. Their findings revealed that they can reduce channels and maintain the classification performance of the given task. Yet, current channel selection methods suffer from poor performance and/or lack a neurophysiological basis [24].

Apart from that, these methods may demonstrate limitations in terms of eliminating irrelevant channels or reducing redundant channels [22]. It is known that the execution of a single task by a participant will trigger functional changes in different brain regions [15]. One could argue that employing all the channels of EEG not only increases the system’s complexity, but also introduces noise, which might decrease the classification performance. Thus, finding an optimal channel selection method is needed to reduce computational complexity and minimize the occurrence of the over-fitting problem, which may be caused by the issue known as the “curse of dimensionality”, in which the error increases as the number of features increases [22].

In addition to the above, the channel selection methods rely on feature extraction methods that extract temporal, spectral, or spatial EEG patterns of signal processing. The feature extraction approaches have been used effectively in improving the EEG classification performance. However, each EEG channel may contain more than one feature, which results in a sharp increase in feature vector space in multi-EEG channels.

Therefore, current research approaches have employed feature selection methods to find the optimal number of features without reducing the EEG channels. Applying feature selection only in multi-EEG channels can be useful in the laboratory because it provides high accuracy due to high spatial resolution. On the other hand, it is not practically effective in home-based applications or daily usage due to the long setup time for electrode placements, which increases computational complexity and affects the comfort level of the user wearing the device. As a result, several methods for obtaining the relevant channels to the source localization of the intended tasks were proposed. The approach for selecting EEG channels could be seen as a feature selection problem. However, the major difference is that channel selection evaluates all features from one channel as a single entity [22].

In terms of EEG features utilized in channel selection methods, Wang [22] adopted EEG spectrogram representations of short-time Fourier transform (STFT) for each channel by treating the data as time–frequency images passed to SVM for emotion classification. Meanwhile, Park [15] and Jing [24] employed raw EEG signals of each channel with the correlation coefficient methods in motor imagery (MI) tasks. In [22], a channel selection method was proposed to select a relevant subset of EEG channels using normalized mutual information (NMI). The method achieved 74.41% and 73.64% accuracy for emotion classification of valence and arousal, respectively, with only eight channels selected. Another proposed method by the authors of [25] used the ReliefF algorithm to find the subset channels corresponding to mental fatigue classification using multi-domain features, and this method succeeded in reducing the number of channels from sixteen channels to eight optimal channels with acceptable accuracy.

The EEG signal is non-stationary but has an event-dependent property for the given task. Therefore, it is important to analyze the changes in the signal with time. Time–frequency feature extraction methods are preferred because they retain the information of both time and frequency. However, time–frequency features such as STFT have high computational complexity while redundant frequency information remains to be solved in real-time STFT applications [26]. The Hjorth parameter proposed in [26] is considered to be a superior alternative to the STFT due to its high ability to extract important information in both the temporal and frequency domains via a simple computing process.

In 1970, Hjorth [27] introduced a set of three time domain parameters to quantify the EEG signal. The Hjorth parameters are often referred to as the normalized slope descriptors due to their ability to be explained by means of first and second derivatives. The first parameter is a mean power value that represents the signal’s activity. The second parameter, called mobility, represents the approximation of the mean frequency. The third parameter is called complexity, which estimates the signal’s bandwidth. Hjorth parameters are computed using variance; thus, they have a low computing cost in comparison to other methods [28]. According to Hjorth, this approach establishes a link between a physical time domain interpretation and the more traditional frequency domain description. Additionally, the time domain context of the Hjorth representation could be advantageous for scenarios requiring continuous EEG analyses for real-life applications. Several studies have successfully employed Hjorth parameters to extract information from various bio-signals, including the detection of the heart rate from the electrocardiogram (ECG) signal, the classification of lung sounds, the classification of the electromyogram (EMG) signal, the diagnosis of hyperactivity (ADHD), epilepsy, and emotion [29,30]. Additionally, Safi et al. [30] reported that EEG Hjorth features improved the detection rate of Alzheimer’s disease.

Efficient channel selection remains a challenging domain in EEG studies for accurately identifying various cognitive states using optimal channels. A range of research have been proposed, each focusing on specific applications and domains related to EEG data. These aim to identify the most informative channels, while simultaneously achieving better computational efficiency and maintaining acceptable accuracy. In Jin’s 2022 study [31], a novel EEG rhythm energy heatmap was proposed, pinpointing optimal channels for each participant and achieving an average classification accuracy of 63.39%. However, the method’s constraint was the variability in the number of channels selected across participants. Moctezuma [32] utilized a multi-objective optimization method with the non-dominated sorting genetic algorithm (NSGA) for an epileptic seizure classification of 24 patients. Notably, it was observed that employing the full set of channels resulted in an accuracy of 0.95. However, the use of only two selected channels increased this accuracy to 0.975%. Wang, 2019 [22], focused on emotion recognition from EEG signals, proposing a channel selection method based on normalized mutual information (NMI). This method drastically reduced channels while maintaining an acceptable accuracy of 74.41% for valence and 73.64% for arousal on the DEAP database. Lokesh, 2022 [33], presented a deep learning model by presenting a hybrid of Convolution Neural Network and Bidirectional Long Short-Term Memory (CNN–BLSTM), focusing on stress levels by utilizing the Physionet EEG dataset. The study found that, with only 19 channels, the accuracy of individual detection can increase up to 99.20%. In Yuxi 2023 [34] study, he proposed a channel optimization algorithm based on sparse logistic regression (SLR), which managed to filter between 75 and 96.9% of channels, resulting in an accuracy increment of 1.65–5.1%. Notably, this method maintained accuracy even with only 2–15 common EEG electrodes across different participants. Hasan, 2020 [35], combined EEG with functional near-infrared spectroscopy (fNIRS) in a hybrid system, selecting only the most correlated channels from each hemisphere and achieving a comparable classification accuracy to existing studies. Pawan, 2023 [36], delved into motor imagery (MI) activity in EEG data, using the Pearson correlation coefficient (PCC) for channel selection. Remarkably, the model selected 14 channels for the sensorimotor area of the brain and achieved maximum accuracies of 91.66% and 90.33% with SVM and K-NN classifiers, respectively. Additionally, Park, 2020 [15], introduced a method for enhancing features for MI classification. Instead of selecting channels based on signal power, the method identified channels through correlation coefficient values, optimizing performance for MI classification tasks.

Compared to other CS methods, correlation-based CS methods have gained researchers’ interest due to their ability to enhance computational efficiency by improving signal-to-noise ratio and offering insights into underlying contextual processes. Their simplicity and adaptability make them especially suitable for diverse applications, ensuring robust results for accuracy and dimensional reduction. For instance, different extensions of correlation-based CS of common spatial pattern (CSP) methods were proposed. The correlation-based channel selection regularized CSP (CCS-RCSP) methods were proposed to find the optimal channels related to motor imagery (MI) tasks using the correlation coefficient [24]. The CCS-RCSP is trained to select the channels that are highly correlated to the MI task. Another extension called filter-bank CSP (FBCSP) was proposed by Park [15] for MI task classification. Additionally, cross-correlation-based discriminant criterion (XCDC) was proposed by the authors of [37] to find the optimal subset channels that are capable of discriminating MI tasks. Another extension of CSP was proposed by the authors of [38] to select internal features and channels based on the difference and the ratio of average L1-Norm for CSP (DRL1 CSP). However, the results of these approaches still provide many channels with the classification task and are specific to the MI task.

A persistent challenge in current channel selection research is the inherent individual differences in brain activity, especially concerning stress tasks. Many studies focus on identifying significant channels for each individual through dependent and/or independent tests. However, this individualization introduces complexities when seeking common significant EEG channels that can be applied across multiple subjects. Overcoming this challenge and pinpointing such universally significant EEG channels could help in the development of real-life applications for stress recognition.

To address the points mentioned above, our key contributions to this work are as follows:

- We introduce an innovative channel selection approach leveraging the correlation coefficient of Hjorth parameters. This method not only identifies, but also ranks universally significant EEG channels across different subjects, ensuring that the classification accuracy remains uncompromised.
- We introduce a new methodology to extract important features from the general optimal channels.
- We validate and compare the effectiveness of the proposed method with the state-of-the-art channel selection methods.

The rest of this paper is structured as follows. In Section 2, the methods and materials, including details of the dataset and data annotations, are described. Section 3 describes the main proposed method for channel selection. Section 4 details the feature set extracted from general optimal channels. Section 5 provides the ML algorithm, the parameters used, and the evaluation matrix. The results of the proposed method and a comparison with existing methods are discussed in Section 6. The detailed discussion of this work follows in Section 7, and the conclusion is given in Section 8.

## 2. Materials and Methods

### 2.1. EEG Dataset

The Dataset for Emotion Analysis using Physiological Signals (DEAP) is a public EEG dataset for emotion recognition [39]. The DEAP comprises data collected at a 512 Hz sampling frequency from 40 physiological channels (32 EEG channels and 8 other physiological channels). In total, 32 healthy subjects participated, with an equal gender distribution of 50% males and 50% females. The EEG data were collected while participants watched selected music videos (40 videos/trials, with each trial lasting one minute) representing the emotion wheel. All participants completed the self-assessment manikin (SAM) [40], rating their arousal levels, like/dislike, valence, and dominance on a scale from 1 to 9. Each subject had 40 trials, with each trial being 63 s long, which included a 3 s pretrial.

The DEAP authors provided preprocessed EEG data. The original EEG data were downsampled to 128 Hz. A band-pass filter ranging from 4.0 to 45.0 Hz was applied to eliminate noise caused by 50/60 Hz line-power and to remove low frequencies (below 4 Hz) resulting from eye blinks. Additionally, artifacts caused by EOG were removed. In this paper, we utilized the preprocessed EEG data provided by DEAP for the mental stress classification task.

### 2.2. EEG Data Annotation

For the 32 EEG channels, data were annotated based on the online self-assessment rating, the SAM scale provided by DEAP, for valence and arousal. In this study, the online self-assessment rating was utilized to distinguish between calming and stressful tasks for each participant, as defined by Equation (1) and as outlined in [41,42]:

(1) stress=(valence<3)∩(arousal>5),calm=(4<valence<6)∩arousal<4)

Valence refers to the pleasantness of the stimulus on a scale of negative to positive, while arousal refers to the intensity level of emotion induced by the stimuli and scales between calm (or low) and excited (or high). A calm state is considered when arousal is low and valence is high. Meanwhile, the stress state is obtained from low valence and high arousal. When the criteria from Equations (1) and (2) were applied to each subject data, seven subjects (with participant IDs: 3, 6, 7, 9, 17, 23, 30) were removed since their data did not contain both stress and calm states. Thus, the rest of the analysis continued with the remaining data of 25 participants.

## 3. Hjorth Multi-Correlation Coefficient

**Figure 1:**
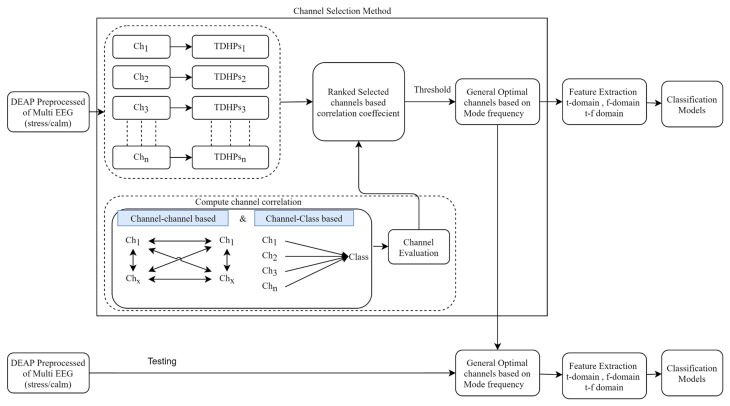
Flow diagram illustrating the proposed method for selecting common EEG channels associated with mental stress detection.

Figure 1 depicts the general flow for selecting channels based on correlation to identify an optimal EEG channel. Essentially, the proposed method favors channels that show strong correlation with class tasks and maintain minimal correlation with each other across various trials. This channel reduction hinges on the premise that specific EEG channels, relevant to the EEG Mental Stress Task (MST), consistently exhibit similar features across all experiment trials when a subject undergoes identical tasks. In contrast, other channels might be less contributive as they are not closely associated with MST. Consequently, we undertook the following procedures to meticulously devise a correlation-centric channel selection approach.

First, we extracted three features of Time Domain Hjorth Parameters (TDHPs), namely activity, mobility, and complexity, from each EEG channel. These parameters offer the advantage of a quantitative evaluation of the EEG signal in the time domain. Table 1 details the TDHPs and their equations. Subsequently, the correlation among these channels was determined based on the statistical measurement of the TDHPs feature set. Next, upon extracting the TDHPs feature vectors for each channel, we applied feature-wise Z-score normalization. This was performed by subtracting each sample value from its feature-wise mean and then dividing the result by the corresponding standard deviation. Finally, we employed the correlation coefficient (CC) method, taking into account the features extracted, to facilitate both channel–channel and channel–class correlations. The subsequent sections delve deeper into the specifics of the CC method.

### Correlation Coefficient Measures

Pearson’s correlation utilizes similarity measurement to find the strength of a linear association between any pair of channels or features in a one-dimensional space. For a given *N* channel, there can be N(N−1)/2 possible pairs for calculating correlations. The pairs of values are considered highly correlated if the correlation coefficient is close to ±1 and uncorrelated if the correlation coefficient is 0 or below a threshold value (i.e., 0.5). The best way to find an optimal projection of the selected channel is to maximize the separation between the two classes. For instance, let us assume that there are two classes of observations (*s*, *c*∈ (stress, calm)). In a one-dimensional feature space, the separation between two classes is defined by the correlation coefficient: let TDHP∈A,C,M represent the features of the activity, complexity, and mobility corresponding to *x*∈(s,c) for classes (stress and calm). The channel–channel-based correlation is computed using the equation below:

(2) $$P_{x}^{\left(S,K\right)}=\frac{1}{\left|l_{x}\right|}\sum_{i=1}^{l_{x}}\frac{cov\left(A_{i}^{S},A_{i}^{K}\right)+cov\left(M_{i}^{S},M_{i}^{K}\right)+cov\left(C_{i}^{S},C_{i}^{K}\right)}{\tilde{A_{i}^{S}}\tilde{A_{i}^{K}}+\tilde{C_{i}^{S}}\tilde{C_{i}^{K}}+\tilde{M_{i}^{S}}\tilde{M_{i}^{K}}}$$

where x∈s,c represents the classes of stress and calm, $l_{x}$ represents the total number of trials of the given class, (S,K) represents the pair channel index, $\tilde{A_{i}^{s}}\tilde{A_{i}^{c}},\tilde{M_{i}^{s}}\tilde{M_{i}^{c}},\tilde{C_{i}^{s}}\tilde{C_{i}^{c}}$ are the standard deviations of TDHPs (activity, complexity, and mobility), and $cov(A_{i}^{S},A_{i}^{K})$ is the covariance of $T_{i}^{S},T_{i}^{K}$, where *T* = *A*, *M* or *C*, and which can be calculated using:

(3) $$cov\left(T_{i}^{s},T_{i}^{k}\right)=\frac{1}{N-1}\sum_{i=1}^{N}\left(T_{i}^{s}-\bar{T_{i}^{s}}\right)\left(T_{i}^{k}-\bar{T_{i}^{k}}\right).$$

$\bar{T_{i}^{s}}$, $\bar{T_{i}^{k}}$ represent the mean of the sample variables $T_{i}^{s}$ and $T_{i}^{s}$, respectively. Then, we computed the main of the two pair channels, as follows:

(4) $$\bar{Ch_{S,K}}=\frac{P_{s}^{\left(S,K\right)}+P_{c}^{\left(S,K\right)}}{2}$$

where $P_{s}^{\left(S,K\right)}$ and $P_{c}^{\left(S,K\right)}$ are the average correlation of the pair of channels of two classes (*s* and *c*) and $\bar{Ch_{S,K}}$ is the main of the two channels. After obtaining the correlation of the channel–channel-based correlation, we computed the class–channel-based correlation using the equation below of (channel–class correlation):

(5) $$p^{\left(s,c\right)}=\sum_{i=1}^{N}\frac{cov(T_{i}^{s},T_{i}^{c})}{\tilde{T_{i}^{s}}+\tilde{T_{i}^{c}}}$$

where T∈TDHPs represents *TDHPs*’ activity, *i* indicates an index of the channel, *s* and *c* represent classes (stress and calm, respectively), and $cov(A_{j}^{s},A_{j}^{c})$ is the covariance of $T_{j}^{s},T_{j}^{c}$. The average of two class correlations of a single channel was calculated as:

(6) $${\bar{Ch}}_{q}=\sum_{T\in TDHPs}p^{\left(s,c\right)},TDHPs=A,M,C.$$

The *F* score was used to estimate the discrimination power of the group of TDHP features since the correlation feature selection depends on a single feature [49]. The purpose of the evaluation function is to precisely find the channel subsets that are highly correlated with the class and uncorrelated with each other. Irrelevant channels with low-class correlations will be omitted. The activation function can be expressed as follows:

(7) $$E_{j}=\frac{K\bar{Ch_{q}}}{\sqrt{K+K\left(K+1\right)K{\bar{Ch}}_{\left(s,k\right)}}}$$

where $E_{j}$ is the significant channels evaluated per independent subject, *k* is the number of channels, $\bar{Ch_{q}}$ is the mean channel–class correlation with (Ch∈S), and the $\bar{Ch_{ch}}$ is the average channel–channel based on inter-correlation.

For general optimal channels among subjects, we counted the frequency of occurrence of each significant channel $E_{j}$ of subjects as the following equation:

(8) $$U_{\left(j,pt\right)}=\left\{\begin{array}{cc} & U_{\left(j,pt+1\right)},\;\;if\;U_{j}=E_{\left(j,k\right)}\\ \; & U_{\left(j,pt\right)},\;\;if\;U_{j}\neq E_{\left(j,k\right)} \end{array}\right\}$$

where $U_{\left(j,pt\right)}$ is the overall unique significant channels among all subjects and pt represents the total unique occurrences of each channel. Then, we ranked them from high to low occurrences and applied a threshold to select the most commonly occurring channels among subjects that appeared in the significant channel sets:

(9) $$G_{j,optimal}=\left\{U_{j}\in U_{\left(j,pt\right)}|\;pt⩾f_{thr}\right\}.$$

$G_{j,optimal}$ represents the general unique significant channels that exist as significant channels on most independent subjects based on the threshold, pt represents the total number of occurrences of each channel, $f_{thr}$ is the threshold, and $U_{\left(j,pt\right)}$ represents the matrix of each unique channel with its repeated number of occurrences. The channel selection based on the correlation coefficient of Hjorth parameters is given by Algorithm 1. These general optimal channels are used for the rest of this paper.
**Algorithm 1:** Channel selection algorithm based on the correlation coefficient of Hjorth’s parameters.**Input:**$N_{p}$ = number of participants, *K* = number of channels$N_{tr}$ = number of trials.Activation $Function\left(E_{i}\right)=\frac{K\bar{Ch_{q}}}{\sqrt{K+K\left(K+1\right)K{\bar{Ch}}_{\left(s,k\right)}}}$.*X* = NULL, subset channels that highly correlated with class and low correlated to other channels.*U* = NULL, is general ranked channel set among participants.**Result**: General Optimal Ranked Channel Set (U)**Method:**
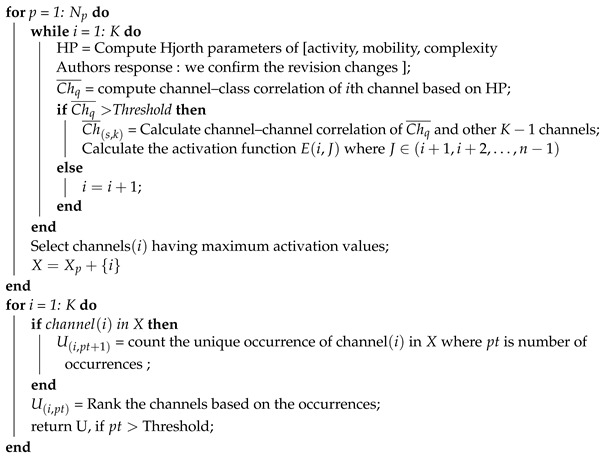


## 4. Feature Extraction

The preprocessed DEAP EEG signals of each participant comprised 40 trials where each trial had 7680 samples (60 s long). In the study by Shon [42], each trial was divided into 16 parts, yielding 480 samples (4 s long) per part. This led to a total of 640 segments per subject (40 trials × 16 segments), which were utilized in this study. Subsequently, we computed the EEG feature extraction of time, frequency, and time–frequency domains from the segmented trial having a time window size of 4 s for the selected general optimal channels proposed by our model. The selected time window size is backed by previous studies which have found that the window size between 3 and 12 s is effective for classifying individuals’ mental status using EEG signals [50,51]. Furthermore, the number of data points within the 4 s is appropriate to demonstrate the stationarity of EEG signals and thus affirm the reliability for achieving channel selection [52,53].

Table 1 presents the features’ descriptions, mathematical equations and the count of each feature per channel used in this study. While previous studies have utilized several time domain features for EEG mental stress and emotion classifications [3,42,51], in this study, we opted to extract multi-domain features. From the time domain, these include: line length, peak-to-peak amplitude, kurtosis, skewness, and Hjorth parameters (activity, mobility, and complexity) of the signal. Concurrently, five features from the frequency domain were extracted based on the relative powers [46] of theta θ (4–8 Hz), low alpha α (8–12 Hz), high alpha α (12–15 Hz), low beta β (15–20 Hz), and high beta β (20–30 Hz). From the time–frequency domain, eight features were derived: six features from the energy of wavelet decomposition coefficient (db4, 6 levels) [11,47], and the spectral entropy of PSD-Welch [48] and Katz’s fraction dimension [43].

A total of 20 features, elaborated in Table 1, were used as a feature set for the optimally selected channels.

## 5. Classification

Both the general optimal selected channels of CCHP and all EEG channels were assessed to distinguish between mental stress and calm emotional state using two classifiers. In the studies of Hasan and Kim [41,42], KNN was employed to classify mental stress and calm state for the DEAP dataset and demonstrated high performance. However, as highlighted in a report by Alex [54], the most common classifier technique applied to EEG signals is SVM. Moreover, in our recent work [55], where we used seven classifiers for an EEG analysis of mental stress, SVM was found to outperform the others. Consequently, in this study, we employed both (Regularized Linear Discriminant Analysis) RLDA, SVM, and KNN to evaluate the proposed method and assess their performance with a minimum number of channels. Herein, RLDA, SVM, and KNN were implemented in Python to classify data into two categories (stress and calm). Table 2 lists the parameter values assigned to each classifier. For each classifier, an independent subject test with 10-fold cross-validations was conducted.

A total of 20 features from multiple domains were extracted from each EEG channel to compose a comprehensive feature vector, as depicted in Table 1. Subsequently, the features of selected EEG channels for each subject were randomly divided into 10 equal subsets based on the 10-fold validation. During each iteration, one unique subset was designated as the test set while the remaining nine subsets were combined to form the training set. We utilized the following metrics to evaluate the performance of the classifiers: precision, recall, and accuracy. Precision denotes the ratio of correctly predicted positive cases to the total predicted positive cases. Recall is characterized as the ratio of correctly predicted positive cases to all observations in the actual class. Meanwhile, accuracy is quantified as the percentage of correct predictions for the test data. The mathematical formulations of precision, recall, and accuracy are enumerated in Equations (10)–(12):

(10) $$Precision=\frac{Tp}{Tp+Fp}$$

(11) $$Recall=\frac{Tp}{Tp+Fn}$$

(12) $$Accuracy=\frac{Tp+Tn}{Tp+Tn+Fp+Fn}$$

where Tp represents the total samples of true positive, Fn represents the false negative, Tn represents the true negative, and Fp is the false positive.

## 6. Result Analysis and Classification

### 6.1. Analysis of Channel Selection

**Figure 2:**
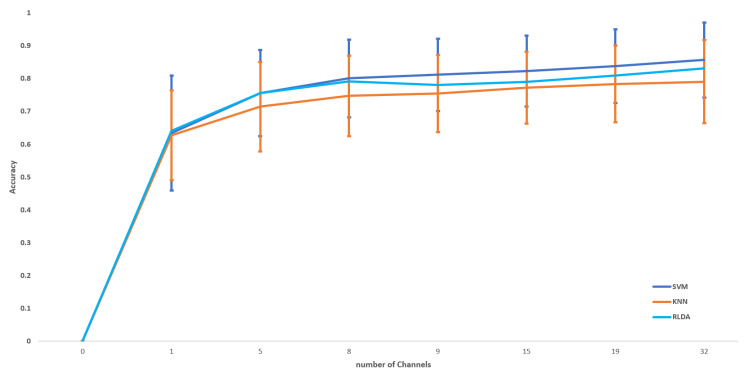
A comparison of mean accuracies based on the number of the most common EEG channels among subjects.

**Figure 3:**
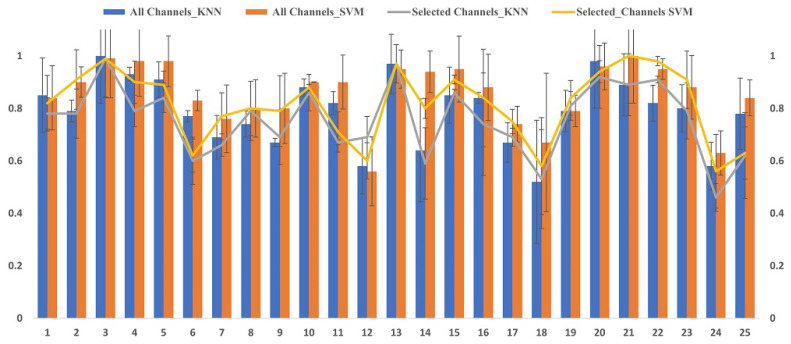
Accuracies and standard deviation for the 10-fold cross-validation per independent subject for 32 channels vs. 8 channels. The bars represent the full EEG channels, while the lines represent the selected significant EEG channels.

**Figure 4:**
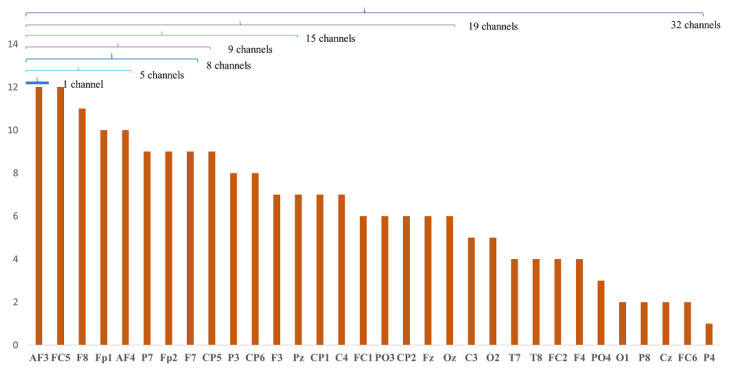
A rank of the common important EEG channels among all subjects.

**Figure 5:**
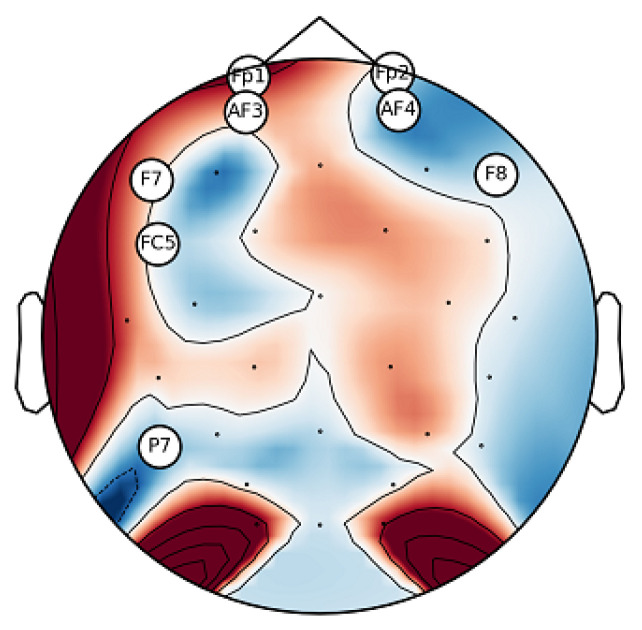
General optimal channels among all subjects that best discriminate mental stress. A circle around the name of each channel represents the significant channels, while the dot symbols are not significant.

**Figure 6:**
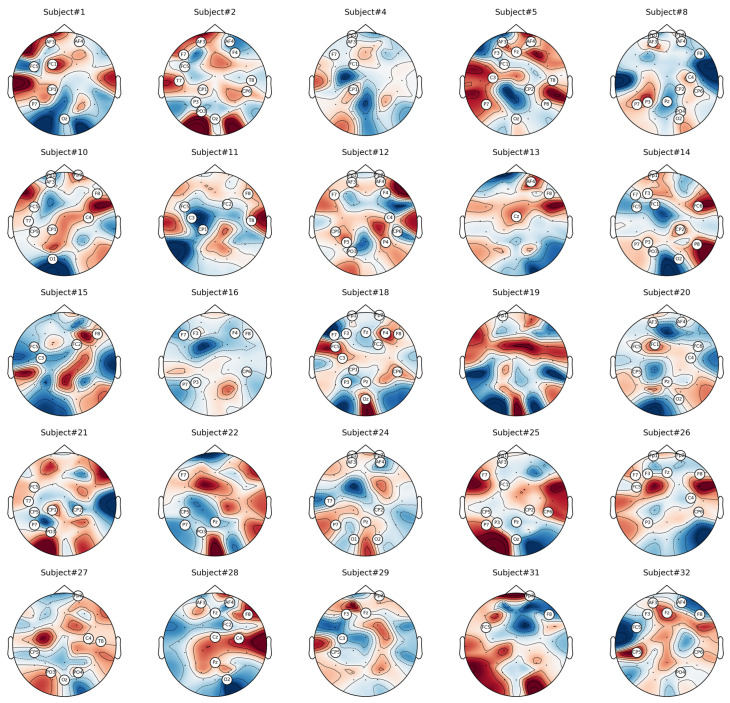
A topographic map of the significant EEG channels in response to mental stress per individual.

The proposed method of CCHP is based on the filter approach which considers an information-based or statistical criterion to provide feedback to the searching algorithms without the classifier’s involvement. The EEG channels were reduced sharply based on the activation function. In particular, for each subject, the proposed CCHP selects EEG channels that are highly correlated to mental state class and are less correlated to other channels within the same class. Selecting relevant channels that are highly correlated to the class (stress/calm) increased performance accuracy. Similarly, the redundant channels were removed by obtaining the low channel–channel-based correlation from the same class. As a result, each subject presented with some important channels that best discriminate mental stress tasks as shown in Figure 2. To find a common channel among all subjects, we ranked the significant channels based on the occurrence of the mode frequency of significant channels as shown in Figure 3, where the high occurrence channels were ranked first, and so on. To determine which set of channels provides an acceptable accuracy, we have compared different sets of ranked channels, 1, 5, 8, 9, 15, 19, and 32 EEG channels, and found that only 8 EEG channels can significantly classify the mental stress state without affecting performance significantly, as shown in Figure 4. Finally, Figure 5 illustrates the selected eight general optimal channels among all subjects that were ranked based on the occurrence of the best subject-independent channels among all subjects as shown in Figure 6.

Figure 2 illustrates the mean accuracies for differentiating between mental stress and calm states using varying numbers of optimally ranked channels. When the entire set of 32 EEG channels was employed to classify mental stress tasks, an accuracy of 85% was observed. This stands in contrast to the 80% accuracy achieved using only eight channels. Notably, utilizing only the top-ranked channel, CH3, yielded an accuracy of 63.4 ± 17.5%. Configurations with 8, 9, 15, and 19 channel sets registered mean accuracies between 80% and 84%. The data reveal a modest enhancement in accuracy when employing the full suite of EEG channels, likely due to the high spatial resolution that covers different brain regions. Nevertheless, with only eight channels, it is possible to secure a satisfactory accuracy, which can help in recognizing the stress states with an optimal number of channels.

Based on insights from Figure 2, we identified the eight channels (‘AF3’, ‘FC5’, ‘F8’, ‘Fp1’, ‘AF4’, ‘P7’, ‘Fp2’, ‘F7’) as the general optimal channels to classify mental stress. To further validate the efficacy of these selected channels, we conducted a two-sample *t*-test. The analysis revealed no significant enhancement (*p* > 0.05) in classification accuracy beyond these eight channels. In conclusion, Figure 5 depicts the location of the general optimal EEG channels on the scalp. These channels were ranked based on the occurrence of the best subject-independent channels and are used throughout the remainder of this paper to recognize the stress/calm mental states of each subject.

### 6.2. Classification Results

We evaluated the proposed channel selection method of CCHP using RLDA, KNN, and SVM. The mean classification accuracy, recall, and precision of each participant were tested in two methods for all channels and with the proposed general optimal selected channels as summarized in Table 3. The classification performance was tested on the two classes (binary classification) of stress and calm states on the EEG data of the DEAP dataset. The classification accuracy of our model using full channels achieved 85.68%, 82.34%, and 79.04% of SVM, RLDA, and KNN, respectively, which outperformed the other stress detection models on the same DEAP dataset. In comparison, our proposed model with eight channels achieved an average classification accuracy of 81.56%, 79.57%, and 75.68% using SVM, RLDA, and KNN, respectively. This suggests that an increased number of EEG channels can lead to a slight increase in accuracy performance.

### 6.3. Performance Comparison of Mental Stress with Existing Methods in DEAP Dataset

To validate the proposed method, we applied the same procedure to the existing methods. Then, we compared the proposed method with the existing methods listed in [22,42] of minimum-redundancy-maximum-relevance (mRMR), short-time Fourier transform with mutual information (STFT + MI), and genetic algorithm (GA). Table 4 summarizes the comparison results of these methods, taking into account the results of three important parameters: the number of selected channels, classification accuracy, and execution time. We further conducted a statistical analysis using the Friedman test for the methods in Table 4 and we found that there was no significance in terms of classification accuracy with Fr = 4.1667 and *p*-value = 0.244. These results confirm the reliability of the proposed method. However, in terms of the number of channels selected, CCHPs obtained fewer optimal channels that are most related to mental stress tasks. The results show that the proposed method yielded the best result in selecting an optimal number of channels within the shortest time compared to the rest of the methods, with eight channels and a 340 ms execution time. Additionally, with regard to classification performance, the proposed method achieved higher results than mRMR but slightly lower than STFT-MI and GA. This difference was attributable to the larger number of channels in GA and STFT-MI. A comparison of different channel selection methods revealed that a minimum number of EEG channels not only reduced the complexity of feature dimensional space, but also preserved the accuracy and reduced the time needed to set up the channels on the scalp. Furthermore, the proposed model results were compared with other related works using EEG signals of the DEAP dataset to recognize mental stress as shown in Table 5. It is evident that our proposed GOC and design paradigm surpassed related works in terms of stress/calm classification with only a minimum number of channels used, with an 81.65% accuracy obtained by eight channels compared to 73.38% of the highest accuracy achieved by Hassan in his study [41]. In addition, Table 6 compares the effectiveness of our proposed channel selection method using different datasets.

## 7. Discussion

The primary objective of our study was to pinpoint the brain regions most sensitive to detecting mental stress states via EEG as we realized that this holds the promise of developing accurate wearable technologies capable of diagnosing mental stress in real-time. Guided by this goal, we introduced an innovative channel selection method based on the correlation coefficient of Hjorth parameters. This method was meticulously tailored to both identify and rank EEG channels in terms of their sensitivity to stress states. A distinctive aspect of our approach involved a comprehensive evaluation, wherein we assessed the effectiveness of various channel groupings, namely 1, 5, 8, 9, 15, 19, and the entire EEG channels. As illustrated in Figure 5, a subset of eight channels (‘AF3’, ‘FC5’, ‘F8’, ‘Fp1’, ‘AF4’, ‘P7’, ‘Fp2’, ‘F7’) as general optimal channels (GOCs) emerged as superior in distinguishing mental stress across a majority of subjects. Notably, these channels predominantly localize in the frontal lobe, as depicted in Figure 5. Our observations align with prior research, which emphasizes the pronounced sensitivity of the frontal brain region to stress [57,58,59]. This sensitivity can be attributed to the Prefrontal Cortex (PFC) of the scalp in both hemispheres. Neurophysiologically, the frontal region’s heightened stress sensitivity can be tied to the Prefrontal Cortex (PFC)’s involvement in executive functions and emotional regulation, its dense dopaminergic pathways affecting stress response, and the influential connectivity between the emotion-centric amygdala and the PFC. These elements collectively highlight the frontal brain’s intricate role in mental stress, offering avenues for optimized EEG channel selection and refining stress recognition systems.

In this paper, we compared the results of the proposed GOC with full EEG channels as shown in Table 3. We observed a slight increase in accuracy when using full channels compared to GOCs, with average accuracies of 85% and 80%, respectively. These results align with previous studies, which demonstrated that full EEG channels could boost accuracy compared to optimally selected channels due to the high spatial resolution provided when using full channels. However, using full channels is not suitable for home-based applications due to computational complexity, extended setup time for EEG electrode placements, and higher costs.

The work presented here provides one of the earliest investigations into identifying and ranking the common important channels for mental stress recognition, as depicted in Figure 4. Here, channels were ranked based on GOC weight across all subjects.

To validate our proposed method, we contrasted it with existing approaches. The proposed GOCs in this study yielded a promising result, taking into account computational complexity using execution time, the number of selected channels, and classification performance as presented in Table 4. These results suggest that our method can determine the common essential channels for real-time EEG stress detection while ensuring relatively high accuracy with merely eight channels. Moreover, the outcomes of our method were derived from EEG data from the DEAP dataset and contrasted with other relevant studies that leveraged the same dataset for stress recognition, as indicated in Table 5. The two investigations led by Hasan [41] and Shon [42] utilized EEG data from the DEAP dataset to differentiate between stress and calm states, as defined by Equation (1), by employing all 32 EEG channels with feature selection methods. Their recorded accuracy rates stood at 71.76% and 73.38%, respectively. Hence, our proposed CCHP model demonstrated superior performance over these other models for mental stress classification. Additionally, Patel, 2023 [56], introduced a novel method for detecting emotional stress using EEG data, classifying stress based on emotional state scores. By applying deep learning techniques, including CONV1D-BiLSTM and CONV1D-BiGRU networks, the research achieved accuracy rates of 88.03% and 75%, respectively.

In comparison to the previous studies, it is worth noting that Peter’s approach involves a distinct method of stress data extraction based on valence and arousal scores from 14 channels. This approach introduces a source of variation, making a direct comparison with the earlier studies somehow challenging. Nevertheless, despite this difference in methodology, Peter’s research managed to achieve notable accuracy rates using deep learning techniques.

While our proposed method has proven to be valuable in selecting commonly relevant channels for mental stress classification, it exhibits some limitations. Firstly, our approach primarily utilizes time domain data. Future research should consider features from other domains. Secondly, the dimensionality of our feature vector remains extensive for real-time applications. Integrating our method with feature selection techniques such as Particle Swarm Optimization (PSO) [60], BAT algorithm [61], genetic algorithm (GA) [42], Whale Optimization Algorithm (WOA) [62], and other heuristic optimization methods might mitigate the “curse of dimensionality” and enhance classification performance. Thirdly, although we achieved high accuracy using the selected channels and SVM with default settings, optimizing SVM parameters remains unaddressed. Future studies should explore this optimization for enhanced outcomes. Moreover, exploring approaches like deep learning with the chosen channels presents substantial potential [63]. Lastly, our research concentrated on features within the cortical activation domain. Investigating other feature types, such as functional connectivity network patterns via graph theory analysis or their integration, could further improve stress detection performance, as highlighted in [53,64].

## 8. Conclusions

In this research, we endeavored to discern between mental stress and calm states by utilizing an optimal selection of EEG channels. We introduced the CCHP method, designed to identify commonly optimal channels across subjects. This approach holds the potential for advancing real-world applications in stress assessment. Our findings underscored that the frontal region of the brain exhibits heightened sensitivity to mental stress. Based on our experimental parameters, the CCHP method ranked channels, with (‘AF3’, ‘FC5’, ‘F8’, ‘Fp1’, ‘AF4’, ‘P7’, ‘Fp2’, ‘F7’) emerging as the optimal channels for differentiating mental stress across participants. Subsequently, we harnessed features from the time, frequency, and time–frequency domains from these eight channels. To train and assess our model, we applied machine learning algorithms, specifically SVM and KNN. Upon comparison with extant methodologies and related studies, our approach demonstrated superior efficacy. Notably, our method successfully differentiated mental stress using only eight channels and achieved a commendable accuracy of 81.56% with the SVM algorithm. The obtained accuracy with the proposed algorithm with eight channels has no significant difference (*p*-value > 2.4) compared to using 32 channels without feature selection. In essence, our work offers a pioneering model that identifies the most prevalent EEG channels capable of detecting mental stress. Such insights are instrumental for the innovation of portable devices tailored for real-time mental stress detection.

## Figures and Tables

**Table 1:** A summary of the feature extraction methods employed in this study.

Domain	Features	Equations	Description	No. Features
Time	Line Length [43,44]	$L\left(n\right)=\sum_{i=1}^{N-1}x\left[i\right]-x\left[i-1\right]$	Called curve length, is the total vertical length of the signal	1
	Kurtosis [45]	$Kurtosis=\frac{\frac{1}{T}\sum_{t=1}^{T}{\left(x\left(t\right)-\mu \right)}^{4}}{\sigma^{4}}$	Shows the sharpness of EEG signals’ peaks	1
	Peak-to-Peak Amplitude	$PTP=pk_{high}-pk_{low}$	Time of EEG signal peaks between the various windows	1
	Skewness [45]	$Skewness=\frac{\frac{1}{T}\sum_{t=1}^{T}{\left(x\left(t\right)-\mu \right)}^{3}}{\sigma^{3}}$	An asymmetry of an EEG signal	1
	Hjorth Parameters [42,45]	Activity=var(x(t))	A variance of the time function;	1
		$Mobility=\sqrt{\frac{var(\frac{dy(t)}{dt})}{Activity(y(t))}}$	A mean frequency or the proportion of standard deviation of the power spectrum	1
		$Complexity=\sqrt{\frac{Mobility(\frac{dy(t)}{dt})}{Mobility(y(t))}}$	Indicates how the shape of a signal is similar to a pure sine wave	1
Frequency	Relative Power [46] of:	$RP=\frac{power(selected\_band)}{power(total\_bands)}\times 100$	Average absolute power of the given band interval	5
	theta (4–8 Hz)			
	alpha (8–12 Hz)			
	sigma (12–15 Hz)			
	low beta (15–20 Hz)			
	high beta (20–30 Hz)			
Time–Frequency	Energy of Wavelet Decomposition Coefficients (db4, 6 level) [11,47]	$Energy\left(E\right)=\sum_{t=1}^{n}x_{t}^{2}$	Measure the square sum of wavelet coefficients of each db level	6
	Spectral Entropy (PSD, Welch) [48]	$SE=-K\sum_{f=4}^{F=45}\bar{PSD}\left(F\right)\times$ $log(\bar{PSD}\left(F\right)),\;k=1.$	Measure the distribution of signal power over frequency	1
	Katz’s Fractal Dimension [43]	$D=\frac{log_{10}\left(n\right)}{log_{10}\left(\frac{d}{L}\right)+log_{10}\left(n\right)}$	Compute the maximum distance between the first point and any other point of the signal time window	1

**Table 2:** Default parameters for classification techniques.

No.	Classifier	Default Value
1	SVM	*C* = 1.0, Kernal = Radial Basis Function (RBF), $1.0\times 10^{-3}$
2	KNN	*K* = 10, distance function = euclidean distance
3	RLDA	solver = eigen, shrinkage = none

**Table 3:** A summary comparison of classification performance for mental stress detection.

Subject ID	All Channels									Proposed Channels								
	**KNN**			**SVM**			**RLDA**			**KNN**			**SVM**			**RLDA**		
	**Precision**	**Recall**	**Accuracy**	**Precision**	**Recall**	**Accuracy**	**Precision**	**Recall**	**Accuracy**	**Precision**	**Recall**	**Accuracy**	**Precision**	**Recall**	**Accuracy**	**Precision**	**Recall**	**Accuracy**
1	0.92	0.7	0.85	0.91	0.69	0.84	0.83	0.73	0.79	0.71	0.62	0.78	0.86	0.65	0.82	0.82	0.72	0.78
2	0.82	0.8	0.79	0.9	0.9	0.9	0.9	0.9	0.9	0.84	0.8	0.78	0.91	0.91	0.91	0.89	0.89	0.89
4	1	1	1	0.99	0.99	0.99	1	1	1	0.99	0.99	0.99	0.99	0.99	0.99	0.95	0.95	0.95
5	0.94	0.93	0.93	0.98	0.98	0.98	0.84	0.84	0.85	0.8	0.79	0.79	0.9	0.9	0.9	0.84	0.82	0.82
8	0.91	0.92	0.91	0.98	0.97	0.98	0.92	0.92	0.92	0.83	0.84	0.84	0.92	0.86	0.89	0.92	0.86	0.89
10	0.79	0.77	0.77	0.83	0.83	0.83	0.63	0.63	0.63	0.6	0.6	0.6	0.62	0.62	0.62	0.62	0.62	0.62
11	0.69	0.71	0.69	0.75	0.71	0.76	0.89	0.8	0.84	0.67	0.68	0.66	0.76	0.71	0.77	0.76	0.71	0.77
12	0.67	0.65	0.74	0.81	0.64	0.8	0.75	0.71	0.76	0.74	0.7	0.79	0.76	0.68	0.8	0.76	0.7	0.76
13	0.55	0.55	0.67	0.78	0.58	0.8	0.84	0.82	0.82	0.55	0.54	0.69	0.76	0.57	0.79	0.55	0.54	0.69
14	0.79	0.73	0.88	0.95	0.67	0.9	0.86	0.71	0.8	0.74	0.76	0.86	0.83	0.65	0.88	0.83	0.65	0.88
15	0.84	0.82	0.82	0.9	0.9	0.9	1	1	1	0.88	0.86	0.87	0.88	0.86	0.87	0.88	0.86	0.87
17	0.78	0.79	0.79	0.9	0.9	0.9	0.93	0.88	0.92	0.81	0.81	0.81	0.9	0.89	0.89	0.9	0.88	0.89
18	0.68	0.65	0.78	0.86	0.78	0.85	0.74	0.71	0.79	0.68	0.65	0.77	0.73	0.67	0.77	0.69	0.66	0.78
19	0.87	0.86	0.87	0.89	0.86	0.89	0.88	0.88	0.88	0.89	0.86	0.88	0.89	0.86	0.88	0.89	0.86	0.88
20	0.71	0.67	0.77	0.81	0.69	0.8	0.82	0.69	0.78	0.75	0.67	0.74	0.77	0.71	0.8	0.77	0.69	0.77
21	0.79	0.77	0.82	0.92	0.88	0.91	0.88	0.88	0.88	0.79	0.77	0.82	0.82	0.8	0.83	0.83	0.8	0.83
22	0.78	0.76	0.83	0.89	0.88	0.89	0.89	0.88	0.89	0.82	0.8	0.82	0.84	0.83	0.84	0.83	0.83	0.83
23	0.87	0.86	0.87	0.93	0.88	0.92	0.88	0.87	0.88	0.87	0.86	0.87	0.87	0.86	0.87	0.87	0.86	0.87
24	0.78	0.73	0.83	0.82	0.73	0.82	0.75	0.72	0.76	0.75	0.71	0.75	0.74	0.72	0.74	0.74	0.72	0.74
25	0.91	0.89	0.91	0.96	0.93	0.94	0.89	0.89	0.89	0.89	0.89	0.89	0.89	0.89	0.89	0.89	0.89	0.89
26	0.89	0.89	0.89	0.97	0.93	0.95	0.91	0.91	0.91	0.89	0.89	0.89	0.92	0.91	0.92	0.89	0.89	0.89
27	0.74	0.75	0.76	0.85	0.83	0.85	0.85	0.84	0.85	0.72	0.72	0.75	0.74	0.74	0.75	0.75	0.75	0.75
28	0.76	0.75	0.81	0.9	0.83	0.88	0.89	0.85	0.88	0.73	0.72	0.78	0.76	0.76	0.81	0.76	0.76	0.81
29	0.87	0.85	0.88	0.92	0.92	0.92	0.89	0.89	0.89	0.85	0.85	0.85	0.89	0.89	0.89	0.89	0.89	0.89
30	0.72	0.72	0.72	0.77	0.77	0.77	0.76	0.76	0.76	0.72	0.72	0.72	0.73	0.72	0.73	0.73	0.72	0.73
31	0.72	0.7	0.77	0.84	0.77	0.83	0.84	0.8	0.82	0.74	0.73	0.74	0.73	0.73	0.73	0.73	0.73	0.73
32	0.85	0.71	0.78	0.89	0.8	0.84	0.84	0.82	0.82	0.57	0.54	0.62	0.6	0.58	0.69	0.67	0.67	0.63
Average	0.76	0.73	0.79	0.85	0.79	0.85	0.83	0.80	0.82	0.71	0.70	0.75	0.80	0.76	0.81	0.77	0.74	0.79

**Table 4:** Performance comparison of the proposed model with other popular existing methods.

Method	No. Channels	Channel Subsets	Classifier	Accuracy	Execution Time
mRMR	11	‘C4’, ‘FC2’, ‘CP6’, ‘Cz’, ‘T8’, ‘F4’, ‘F8’, ‘P4’, ‘Fz’, ‘FC6’, ‘Pz’	SVM	0.80 ± 0.12	1.42 s
			KNN	0.74 ± 0.12	
			RLDA	0.79 ± 0.13	
STFT + MI	15	‘AF3’, ‘F7’, ‘FC5’, ‘P3’, ‘P7’, ‘Pz’, ‘O2’, ‘P4’, ‘FC6’, ‘Fp2’, ‘FC1’, ‘CP2’, ’C4’, ‘F4’, ‘Fz’	SVM	0.82 ± 0.11	4.46 s
			KNN	0.74 ± 0.12	
			RLDA	0.80 ± 0.14	
GA	13	‘O2’, ‘O1’, ‘PO3’, ‘AF3’, ‘P4’, ‘P8’, ‘F8’, ‘P7’, ‘C4’, ‘CP5’, ‘Pz’, ‘FC5’, ‘Fp2’	SVM	0.82 ± 0.12	1 h 3 min 34 s
			KNN	0.76 ± 0.13	
			RLDA	0.81 ± 0.13	
Proposed	8	‘AF3’, ‘FC5’, ‘F8’, ‘Fp1’, ‘AF4’, ‘P7’, ‘Fp2’, ‘F7’	SVM	0.81 ± 0.11	0.34 s
			KNN	0.75 ± 0.12	
			RLDA	0.79 ± 0.12	

**Table 5:** Performance comparison of stress detection with related works using EEG signals in the DEAP dataset.

Author	Method	EEG Channels	Accuracy/Class
Shon [42]	Genetic Algorithm-Based Feature Selection	32	71.76% (Stress/Calm)
Hasan [41]	Boruta-based k-NN feature selection	32	73.38% (Stress/Calm)
Patel [56]	CONV1D + BiLSTM	14	88.03% (Valance–Arousal Score Level)
	CONV1D + BiGRU	14	75% (Stress/Calm)
Proposed	Full Channels SET + SVM	32	85.68% (Stress/Calm)
	CCHP + SVM	8	81.56% (Stress/Calm)

**Table 6:** Comparison of channel selection method on different datasets.

Dataset	Channels	No. Channels	Accuracy
EDMSS	Total	7	77.31%
	Selected	5	75.23%
DEAP	Total	32	85.68%
	Selected	8	81.56%
SEED	Total	62	83.21%
	Selected	8	80.31%

## Data Availability

Raw EEG data of EDMSS’ dataset can be obtained by writing a formal email to Fares Al-Shargie. http://bcmi.sjtu.edu.cn/home/seed/index.html, https://www.eecs.qmul.ac.uk/mmv/datasets/deap/.

## Abbreviations

The following abbreviations are used in this manuscript:

CCHP	Correlation Coefficient of Hjorth Parameter	A novel approach used to determine the correlation between Hjorth parameters for EEG channel selection
DEAP	Dataset for Emotion Analysis using Physiological Signals	A widely used dataset for emotion recognition tasks
CS	Channel Selection	Technique used to select the most informative EEG channels
GOCs	General Optimal Channels	The EEG channels that are found most effective across different subjects
PFC	Prefrontal Cortex	The front part of the brain, known to be responsive to stress and involved in cognitive functions
